# Restoring trust in vaccination: listening to patients and acknowledging Post-Acute COVID Vaccine Syndrome

**DOI:** 10.3389/fmed.2025.1688170

**Published:** 2025-11-28

**Authors:** Matthew Halma, Joseph Varon

**Affiliations:** 1 Open Source Medicine OÜ, Tallinn, Estonia; 2 Independent Medical Alliance, Washington, DC, United States

**Keywords:** Post-Acute COVID-19 Vaccination Syndrome, Long COVID, Post-Acute COVID-19 Syndrome (PACS), spike protein, adverse event following immunization (AEFI)

## Abstract

The National Academies of Science, Engineering, and Medicine (NASEM) has defined Long COVID as “an infection-associated chronic condition (IACC) that occurs after SARS-CoV-2 infection and is present for at least 3 months as a continuous, relapsing and remitting, or progressive disease state that affects one or more organ systems.” This definition puts the experience of the patient primary, where the decisive factor for diagnosis is a persistent health problem after COVID-19 infection. Ongoing work aims to characterize the biological signature of both Long COVID and Post-Acute COVID-19 Vaccination Syndrome (PACVS), clinicians and researchers are faced with heterogeneous diseases that are not easily captured by a single biomarker. Candidate biomarkers establish spike protein persistence, either through detection of full length spike, the S1 subunit of spike protein, or anti-spike protein antibody positivity. Additionally, to rule out viral reservoirs or active infection as an explanation, anti-nucleocapsid antibody, a hallmark of COVID-19 infection not present in the vaccine, should be negative. Other candidate biomarkers include detection of vaccine sequence mRNA, or sequence differentiation of viral from vaccinal spike through mass spectrometry. Despite candidate biomarkers, medicine is far from a definitive diagnostic test. Lack of diagnosis has created negative experiences for patients and strengthened vaccine hesitancy. An open acknowledgement of vaccine risks is vital to restoring trust in science and medicine and ensuring those injured have access to the care they need.

## Introduction

In 2024, the National Academies of Science, Engineering, and Medicine developed a working definition of Post-Acute COVID-19 Syndrome (PACS) or Long COVID (LC) syndrome^1^, defined as “an infection-associated chronic condition (IACC) that occurs after SARS-CoV-2 infection and is present for at least 3 months as a continuous, relapsing and remitting, or progressive disease state that affects one or more organ systems” (1). This definition has been controversial, as critics maintain that it is too liberal and too based on patient self-reporting (2). On the other hand, many PACS patients are acutely aware of marginalization by healthcare providers, as many have to see multiple doctors before receiving care (3), a finding which parallels PACVS patient experience (4).

One often used justification is the current paucity of biomarkers that can be used to diagnose patients. A similar case exists with Lyme disease, where the first Lyme cases appeared in 1975, in Connecticut (5). It was not until 1982 and 1983 that the etiological agent of Lyme disease, *Borrelia burgdorferi,* was identified (6, 7), and diagnostic tests became available soon after (8). Despite a test for an etiological agent linked to the disease and fulfilling Koch’s postulates for Lyme disease etiology (9), patients still face skepticism, even today, when they attribute their symptoms to Lyme disease and have molecular test results (10).

While the etiological agent of PACS is (by definition) acute COVID-19 infection, several hypothesized mechanisms, or a combination of different mechanisms, may explain PACS persistence. One prominent explanation is the viral persistence hypothesis, which postulates that the SARS-CoV-2 virus, or components or fragments of it, remain in the body after the acute infection. This explanation has some support, as circulating viral antigens have been detected in PACS patients’ blood, and can even recreate similar physiological changes during PACS in an animal model (11, 12). While there need not be a singular etiological agent to explain all pathological symptoms, and the high degree of patient heterogeneity points to multiple agents and multiple mechanisms, several candidate agents have been proposed.

One prominent possible agent is the spike (S) protein, expressed both during Acute COVID-19 infection and immediately following COVID-19 vaccination, making it a similar, albeit not identical, etiological agent for both PACS and PACVS. Given the apparent similarity of symptoms between PACS and PACVS, a shared etiological agent is a parsimonious explanation. PACVS and PACS both share fatigue, post-exertional malaise (PEM), and brain fog as common symptoms, occurring in a majority of patients for both PACS and PACVS. Other non-specific biomarkers (besides spike protein) identified thus far may be useful at differentiating the physiological signature between PACS and PACVS (13), though sample sizes are low and the populations are highly heterogenous.

## PACS and PACVS biomarkers

An increasing literature base has determined statistically significant biological markers related to Long COVID (PACS) and Post-Acute COVID-19 Vaccine Syndrome (PACVS). These studies illustrate that chronic viral antigens, viral proteins in the extracellular vesicles, and erroneous autoimmune reactions could be useful objective indicators of ongoing pathophysiology. Several reports have indicated that the full-length spike protein and its S1 subunit are still detected many months following acute infection. Craddock et al. (14) found soluble and extracellular-vesicle-associated spike in plasma of Long COVID patients through 12 months of initial illness, and reported persistent antigenic stimulation. On a similar note, Yonker et al. (15) reported higher levels of full-length spike in adolescents who presented with post-mRNA-vaccine myocarditis, indicating that spike antigenemia may also be used as an indicator of post-vaccination adverse events.

Recent reports by de Miguel-Perez et al. (16) indicate that spike, Nucleocapsid, and other viral elements in extracellular vesicles are correlated with dysfunction of multiple organs and poor clinical outcomes. EV-associated viral proteins are stable, biologically active, and may represent active intracellular expression or slow clearance. Mantovani et al. (17) found anti-G-protein-coupled receptors (GPCRs) and anti-Ras-related molecules autoantibodies in patients with persistent post-COVID-19 vaccination symptoms. These results propose an immune-based endotype of PACVS which can be distinguished from classical PACS. The study by Krishnamachary et al. (18) showed a positive relation between EV-induced endothelial apoptosis and the severity of COVID-19, which aligns with the importance of the vascular injury cues in chronic post-COVID-19 conditions. The biological heterogeneity of PACS is also supported by complement activation fragments, cytokine increases, and microvascular dysfunction markers that are found in other studies.

## Role of S1 in PACS and PACVS

SARS-CoV-2 S1 subunit has been suggested as one of the primary causes of the long-term biological malfunctioning in both Long COVID (PACS) and post-vaccination symptomatology (PACVS). S1 can act independently after being cleaved and released into circulation, unlike the full spike protein. A number of studies have shown that S1 interacts with ACE2 and leads to its internalization, that causes endothelial dysfunction, mitochondrial damage, oxidative stress, and a decrease in vascular homeostasis (19, 20). These endothelial alterations play a role in microvascular instability, release of inflammatory cytokines, abnormal vasoregulation, and a pro-thrombotic state-mechanisms that are very consistent with the multi-system symptom patterns that are frequent with Long COVID. Notably, it has only recently been discovered that the S1 subunit, either in extracellular compartments or in the soluble protein state, persisted in the circulatory system long after acute infection (14). This continuity gives a biological explanation of long-term endothelial and inflammatory activation.

S1 also has strong neuroimmune effects in addition to vascular injury. It has been experimentally demonstrated that S1 has the potential to trigger microglia and mast cell activation, impair the blood–brain barrier, and activate innate immune receptors like TLR4, triggering further neuroinflammation even in the absence of a replicating virus (21). These processes can be the basis of the cognitive impairment, autonomic disequilibrium, sensory, and fatigue that is so common in PACS. The same pathways apply to PACVS since vaccine-encoded spike contains the S1 domain, and shedding of S1 after the expression of mRNA constructs has been reported. In fact, spike and S1 circulating levels were found to be measurable in post-vaccinated subjects with myocarditis (15), which confirms the idea that post-vaccination antigen persistence and S1-induced dysregulation can be observed in both diseases. Collectively, these results make the S1 subunit a biologically marked and biologically plausible participant of long-term post-COVID symptomatology, which is why it should be considered a candidate biomarker.

## Toxic biological effects of the SARS-CoV-2 spike protein

Several publications have proven that the SARS-CoV-2 spike protein, with its subunit S1, has inherent biological toxicity that can lead to long-term post-acute effects. Lei et al. (19) demonstrated that spike itself is enough to induce impaired endothelial performance via ACE2 downregulation and the initiation of inflammatory signaling, which Gao et al. (20) supported by demonstrating that spike-mediated ACE2 internalization is a cause of endothelial injury in the absence of viral replication. Chang et al. (22) also found that spike contributes to chronic oxidative stress and vascular dysfunction, and is a mechanistic explanation of how microvascular symptoms of Long COVID can be seen. Moreover, spike-related extracellular vesicle-associated spike proteins have been demonstrated to cause endothelial apoptosis; Krishnamachary et al. (18) discovered that EVs with spike were strongly correlated with COVID-19 severity and vascular injury.

The S1 subunit is also pro-inflammatory and cytotoxic. The results of Kumar et al. (23) show that S1 enhances endothelial injury and inflammation, and the impact thereof can be aggravated by hormonal factors. Theoharides and Kempuraj (21) demonstrated that mast cells and microglia are activated by spike, which is a mechanistic explanation of neurological symptoms in PACS. In a similar manner, variant-associated and systemic toxicity of spike was described by Moghaddar et al. (24) and Almehdi et al. (25), which included the impact on vascular, immune, and inflammatory pathways. Letarov et al. (26) also suggested that free S1 particles could remain and play a direct role in the pathology of inflammation. These studies, in combination, offer the mechanistic contribution of spike toxicity in PACS and PACVS.

## Spike expression after adverse vaccine effects

The existence of SARS-CoV-2 spike protein in people with adverse events post-mRNA vaccination has been reported several times, which supports the biological plausibility of spike-mediated post-vaccine symptoms. MRNA vaccines have been linked to neurological complications, and spike expression has been detected in affected tissues, as indicated in a recent series of case reports published in the Journal of Clinical Neuroscience (27), as well as in another clinical report of similar outcomes (28). spike protein expression in the cutaneous lesions that cause dermatological manifestations after mRNA vaccination has also been shown (29), also demonstrating multi-organ expression of spike in post-vaccination response.

Patterson et al. (30) have provided one of the most detailed analyses and proved persistent S1 spike protein in CD16 + monocytes up to 245 days in SARS-CoV-2-negative individuals with post-COVID-19 vaccine syndrome (PCVS), which suggests the existence of an active antigenic response even after the vaccination. Furthermore, the American College of Cardiology stated that the presence of circulating full-length spike protein was detected in the cases of post-mRNA vaccine myocarditis among adolescents, which proved the existence of a mechanistic association between spike expression and cardiac inflammation (15). The available evidence of immunopathology provided by Federico (31) makes the same point with a wider range of immune activation that is related to the presence of fragments of spike in the blood and highlights the biological significance of ongoing or dysregulated spike expression after the vaccine. All these studies together imply that in uncommon instances of adverse vaccine reactions, the spike protein can appear in the blood or in the tissues and could cause clinical symptoms.

## Suggested biomarker framework of Long COVID (PACS) and Post-Vaccine COVID-19 Syndrome (PACVS)

The lack of a standard biomarker construct that can be used to distinguish (a) previous infection, (b) vaccine-induced responses, and (c) ongoing pathogenic processes has remained a long-standing drawback of the assessment of post-acute SARS-CoV-2 sequelae. We suggest an evidence-based biomarker panel combining serologic, antigenic, EV-associated, proteomic, and molecular testing. The framework is based on current peer-reviewed papers that show continuous spike antigenemia, EV-associated viral proteins, GPCR/RAS-associated autoantibodies, and proteomic evidence which discriminates between vaccine-encoded spike and viral spike.

### Serologic biomarkers: anti-spike (anti-S) and anti-nucleocapsid (anti-N) antibodies

Anti-S and anti-N antibodies are clinically useful in giving contextual information on previous exposure to an antigen. Anti-S are indicative of infection as well as vaccination, whereas anti-N are usually specific to previous infection. The presence of semi-quantitative assays of total antibodies (IgG + IgM + IgA) has been found to be correlated with long-term symptoms in both vaccinated and unvaccinated people with Long COVID (32). Serology cannot diagnose PACS or PACVS, but it provides information on whether persistent symptomatology occurs after infection, vaccination, or both. Serology, when used together with antigenemia or EV-spike testing, can be used to build up an exposure timeline, as both syndromes may overlap in their clinical presentation.

### Spike antigenemia: full length spike and S1 subunit

There has been growing evidence in favor of the contribution of the continuing circulation of spike protein-and especially the S1 subunit, to the pathophysiology of Long COVID and vaccine-related adverse events. Yonker et al. (15) were able to detect full-length spike in adolescents who had post-mRNA-vaccine myocarditis, and Craddock et al. (14) measurably found persistence of soluble and EV-bound spike up to 12 months in patients with Long COVID. Special attention is paid to persistent S1 because of its ability to cause endothelial dysfunction, impair mitochondrial energetics, trigger inflammatory signaling pathways, and divert endothelial cells to glycolytic metabolism (19, 20). These direct cytopathic effects offer a mechanistic explanation of the quantification of full-length spike and its S1 cleavage product in plasma by immunocapture analyses or specific proteomic methods. The persistence of the spike antigenemia weeks to months following the initial exposure is indicative of persistent protein production, failure to clear, or depot release of the antigen in the tissue reservoirs.

### Viral proteins associated with extracellular vesicles

EVs are a separate compartment of biomarkers that have a high level of mechanistic significance. De Miguel-Perez et al. (16) demonstrated that EVs with SARS-CoV-2 spike, N, and other viral proteins are connected with the dysfunction of multiple organs and worse outcomes during severe COVID-19. The evidence of EVs eliciting endothelial apoptosis was also shown by previous studies using hospitalized COVID-19 patients (18). EV-associated spike is an additional signal to soluble antigenemia and possibly an even more reliable substitute for active antigen presence. The identification of EV-bound spike by size-exclusion isolation, then, combined with ELISA or mass spectrometry, may therefore be used as a second-line biomarker in patients who have ongoing symptoms despite a negative PCR test.

### RAS-related molecules and GPCR autoantibodies

Recent literature indicates an autoimmune involvement in a fraction of PACVS patients. According to Mantovani et al. (17), people with persistent, post-vaccination symptoms have autoantibodies against GPCRs and RAS-related molecules. These immunologic imbalances are likely to be involved in dysautonomia, vascular instability, and systemic inflammatory responses that are often characterized in PACS and PACVS. Not being spike persistence specific, these autoantibodies can be used as adjunct biomarkers that identify immune-mediated endotypes.

### Proteomic distinguishing between viral vs. vaccine-encoded spike

It can be technically achieved with specific LC–MS/MS analyses of peptide profiles, since mRNA vaccines encode a prefusion-stabilized spike protein with the mutations K986P and V987P (2P) not present in the viral sequence. The 2P region consists of protein forms in a manner that allows the unambiguous identification of spike origin through proteotypic peptides. Several studies have shown that immunocapture and mass spectrometry can be utilized in the quantification of clinical samples of viral proteins (33, 34). Furthermore, peptide and glycopeptide libraries made of variants can also improve source attribution.

## Mass-spectrometry differentiation of vaccine vs. viral spike protein

A big diagnostic question for both PACS and PACVS is whether the persistent spike protein is a result of previous infection, or it has been expressed as a result of vaccination. This difference can be analyzed analytically by means of specific mass spectrometry. The mRNA SARS-CoV-2 vaccines have two stabilizing proline substitutions, the 2P mutations, K986P and V987P, present in the spike S2 subunit that are absent in wild-type viral spike. Such replacements of amino acids form distinctive proteotypic peptides that could be specifically identified in LC–MS/MS, and therefore, one could simply assign the spike origin (35).

Targeted mass spectrometry using immunocapture has been confirmed to be effective in the quantification of the SARS-CoV-2 structural proteins. As Pierce-Ruiz et al. (33) have shown, immunocapture and the subsequent use of isotope dilution mass spectrometry are reliable in the quantification of the viral antigens in clinical samples. Likewise, Nikolaev et al. (36) also reported the presence of several spike- and N-derived peptides detected with high sensitivity by LC–MS/MS, which demonstrates the possibility of identifying the viral proteins of the patient-derived specimens. Even more recently, Sutton et al. (34) directly measured vaccine-produced spike with targeted LC–MS/MS and found that the spike protein was directly measurable following expression upon vaccination.

Also, Suddhapas et al. (37) created peptide panels that discriminate between variants of SARS-CoV-2 using LC–MS/MS, which serves as a methodological precedent for differentiating between spike isoforms using the peptide signature. Application of this method to PACS/PACVS: Peptides of the 986–987 region would unambiguously detect vaccine-derived spike, whereas peptides of unique infectious variants of the virus (e.g., Delta, Omicron) would detect infection-derived spike.

Immunocapture to enrich, targeted MRM/PRM transitions to quantify, and peptide selection of mutation-containing regions make it possible to have a robust, reproducible, and clinically scalable biomarker assay. This technique offers a molecular-based endpoint of defining whether chronic spike antigenemia occurs as a result of prior infection with SARS-CoV-2 or as a result of continued expression or slower clearance of vaccine-induced spike.

## PCR identification of persistent vaccine mRNA

Besides antigen-based and proteomic biomarkers, PCR tests have the potential to be applied in identifying persistent vaccine-derived mRNA in suspected PACVS. The BNT162b2 (Pfizer-BioNTech) vaccine has a number of sequence elements that are not similar to the SARS-CoV-2 viral genome and can be identified specifically by molecular detection. It is particularly noteworthy that the vaccine has a modified 3′ untranslated region (3’UTR) of hybrid elements based on human 12S rRNA (mtRNR1) and synthetic AES sequences that the native SARS-CoV-2 RNA does not have. Such distinctive motifs have high specificity of primer-binding sites that can be used in qPCR assays to identify the presence of residual vaccine mRNA transcripts (38).

Furthermore, the BNT162b2 construct expresses the prefusion-stabilized spike protein with 2 proline replacements (K986P and V987P) (the 2P mutations). PCR primers that have been designed to target this engineered region can further distinguish between vaccine mRNA and viral RNA since the genome of SARS-CoV-2 does not have these replacements. Camperi et al. explain in detail how analytical techniques, such as qPCR, can be customized to be specific to these distinctive features of the sequence, with the intention of characterizing the vaccine construct (39).

When vaccine mRNA is long-lived and outlives its degradation duration, these sequences would be detected, and this result would suggest that there is still a potential to be translated, and the presence of a pathway to keep producing spike. Despite the fact that the clinical relevance of persistent vaccine mRNA is under study, PCR-based detection is a complementary molecular biomarker that can aid in the differentiation of PACVS and PACS, especially when spike antigenemia or S1 positivity occurs, and the issue of source attribution is critical (40).

## Mechanisms involving the production, biodistribution, and persistence of spike protein

Infection and vaccination differ significantly in their effect on the host’s biological system. From the lens of mechanism, and focusing on spike protein, systemic infection produces spike protein in a more distributed fashion, where infected cells produce SARS-CoV-2 virions, with spike (S) protein and nucleocapsid (N) protein composing the outer shell of the virion. These are released via exocytosis or cell lysis. In the context of infection, the spike protein is either bound to the virion or may be cleaved at the Furin cleavage site (FCS), in which case free S1 subunits will be circulating. spike protein may also be encountered by the body in the context of antigen presentation on the cell surface for immune recognition. The case of circulating S1 subunits is most likely the context of vaccination, where vaccinal mRNA enters cells through the fusion of the modified mRNA-containing lipid nanoparticle (LNP) to the cell membrane, which is then expressed and displayed on the cell surface. Free spike protein is observed in both subsets of PACVS and PACS (15), so the spike can be unanchored. In the case of post-vaccination myocarditis, the level of total spike protein appears to have greater power to discriminate post-vaccination myocarditis cases from healthy vaccinees than levels of the S1 subunit of the S protein (15).

In the case of vaccination, the spatio-temporal profile of spike protein expression is, in theory, much more localized and limited in time than in the case of infection. During the vaccine rollout, it was thought that the spike protein would only be produced spatially near the injection site and in more distal lymph nodes and would only be produced for a short duration (41). In some cases, arguments for the restricted time course of spike protein were based on arguments from analogy with unmodified mRNA, despite the chemical modifications to the mRNA for enhanced translational efficiency and resistance to breakdown (42).

Biodistribution studies in animals, as well as fluid samples, tissue biopsies, and autopsies, have revealed a greater degree of biodistribution of spike protein than initially anticipated, and also significantly longer persistence times, with spike protein being observed 245 days post vaccination in PACVS patients (30). These findings mean that there is a longer tail to the kinetic curve for spike protein, and also may be more spatially spread out, with spike protein being observed in a wide variety of organs after vaccination.

Spike protein distribution in the case of infection is governed by tissue tropism of the virus for different tissues and cell types, which explains the susceptibility of those cell types to SARS-CoV-2 infection. By comparison, LNPs may be more promiscuous and distribute their encapsulated mRNA(s) to any cell type, so long as the LNP comes into contact. The production of encoded protein is greater in using n1-methyl-pseudouridine (43), the nucleoside modification adopted by Pfizer (BNT162b2) and Moderna (mRNA-1273) (42).

## Barriers in causality assessment, reporting, and institutional response

These findings have contradicted the previous assumption that vaccine adverse effects would be restricted to the 2 weeks after immunization, after which, it is more difficult to assign causality under the WHO criteria (44, 45). There may still be ongoing spike protein production, contributing to pathological processes from the spike protein itself (46–48), or potential immune dysregulation. While clinicians have some standard procedures in place for dealing with drug adverse reactions, few are aware of vaccine adverse events reporting requirements as per the 1986 Vaccine Safety Act (49), and in some cases, doctors have been dissuaded from submitting reports to the Vaccine Adverse Events Reporting System (VAERS) (50).

Bellavite et al. (44) identified several shortcomings of using the WHO causality algorithm for vaccines, where it may potentially ignore or disqualify a causal relationship. If there is another factor present which can explain the symptoms, this essentially disqualifies causal assessment in that case. Importantly, it is vital to note the similarity in presentation between PACVS and PACS. Given the similarity in symptoms and also the potential for confounded etiology, besides the COVID-19 unvaccinated and the small minority of people who remain uninfected from SARS-CoV-2, most people have been exposed to both SARS-CoV-2 and SARS-CoV-2 vaccination, making it difficult to assign clear causality to a single entity, especially with the possibility of repeated asymptomatic infections (51).

Though the temporal proximity between the vaccination and the occurrence of the symptom might help in the distinction between PACVS and PACS, this relationship should be interpreted with caution because the short-term post-vaccination reactogenicity (e.g., fatigue, fever, myalgia) is well-known in the first 48–72 h after mRNA vaccination (52, 53). VAERS is a passive surveillance program that tries to identify early warning signals of safety, but is underreported, not completely data-filled, and lacks verification procedures (54). The reports made to VAERS do not prove causality: the reports are initial indications that ought to be formally epidemiologically reviewed. Despite what critics imply (55), fraudulent reports (punishable by fines or imprisonment under federal law) are rare and usually removed, representing a negligible proportion of total reports (56).

While either patients or medical professionals can be mistaken, causal relationships are more likely to be ruled out for vaccination than for another perturbation. In the clinical setting, it would typically be considered improper to rule out a suspected relationship with a drug, especially if there is a mechanistic basis and the relationship has been established beforehand (57).

While PACVS is currently much more acknowledged, it was not always this way. The road to acknowledgement has been long and fraught, and faced institutional neglect and hostility the entire time. Subjects injured during COVID-19 vaccine trials have experienced significant difficulty to receive compensation for medical expenses (58). Official messaging typically minimized risks and the Biden administration was pressuring social media companies to remove content promoting what it defined as “vaccine misinformation,” which included genuine misinformation alongside scientifically supported statements, disputed statements, and patient experiences (59). Restrictions on online speech likely contributed to a suboptimal COVID-19 response (60), and likely contributed to marginalization of PACVS patients (13).

## Recognition, diagnosis, and research needs

Given these challenges, PACVS patients need concrete support and resources, not the vague bromides which they were given under Peter Marks (61). The vaccine-injured have been tasked with funding their own research after taking a product that they were encouraged to take, and in some cases required to take as a condition of their employment. This state of affairs is unacceptable.

As a possible response to the pandemic (62), vaccine uptake has dropped significantly, and given the ubiquity, a significant number of people are either directly affected by PACVS (63) or know somebody with PACVS.

To reduce gaslighting, medicine can adopt a similar approach for PACVS as it has for PACS, though real-world adoption remains slow. We translate the NASEM Long COVID diagnostic guidelines (1) for the context of PACVS, providing the following definition:

“Post-Acute COVID-19 Vaccination Syndrome (PACVS) is a vaccination-associated chronic condition that occurs after SARS-CoV-2 vaccination and is present for at least 3 months as a continuous, relapsing, and remitting, or progressive disease state that affects one or more organ systems.”

Adopting this definition may reduce stigma and gaslighting by practitioners, though it takes some time for cultures to shift. The doctor-patient relationship can often be paternalistic in practice. For the case of vaccines, the education that doctors receive does not provide any coverage of vaccine adverse events or how to report or treat them. Researchers are trying to figure out the cause and treatments for vaccine injuries, but work with limited resources and are often frozen out of institutions or professional collaborations (64).

Academia remains very pro-vaccine, and rightfully so, given the role that vaccines play in preventing infectious diseases, but this enthusiasm may come with a blindness to their potential adverse effects. Constant messaging about “vaccine misinformation” may make doctors less charitable to the PACVS patient before them (65).

Science and medicine need to collaborate fast. We recommend that PACVS be added as a diagnostic code for systems using the ICD-10 or ICD-11 system. This is a genuinely novel disease, as it only existed after the advent of COVID-19 vaccines. Furthermore, all vaccines can have adverse events associated with them, so it is important to recognize these as well.

For research, it is important to develop biomarkers, which requires biobanking samples from individuals and associating them with their clinical state and trajectory. Susceptibility factors must be identified to understand the disease mechanism (66), and to guide the future provision of medical exemptions and other preventative mechanisms.

Importantly, clinical trials must be run, and money needs to be put aside for this explicitly. This will allow for the creation of therapeutic protocols, for which there are currently few with an evidentiary basis, and many products on the market with limited evidence. PACVS patients often have tried many different therapies with limited success (13). In order to restore trust, begin by listening to the patient’s experience.

## Conclusion

While the COVID-19 vaccines prevented many infections and deaths from COVID-19, they resulted in a small percentage of individuals developing acute and chronic complications. Those with post-acute complications face a crisis in a lack of recognition, which results in their inability to access care and deprioritizes research, as they are not considered a source of disease burden.

The disease burden from PACVS is potentially large, with credible estimates of between 0.2 to 0.9% of COVID-19 vaccine recipients developing the condition (63). Individuals with PACVS need a means to access care, so a new diagnosis must be added for this novel condition. Over the past few years, a more official definition and terminology have solidified around PACVS, and it is currently acknowledged by many medical and scientific authorities. This acknowledgement comes after years of active marginalization by medical authorities. Social media companies were pressured by the US government to remove support groups for post-vaccination complications, under the justification of removing vaccine-related misinformation, which, in many cases, was factually accurate or personal testimony (67).

These initial actions have created an unacceptable delay in recognition and have actively marginalized and gaslit those PACVS patients. Likely, given the high heterogeneity of biomarkers in PACVS (and PACS, for that matter), the development of a rigorous diagnostic test will take time. Until then, it makes most sense to define PACVS using the NASEM definition of PACS, with adaptation for the different etiology. A formal diagnostic framework of PACVS can be supported by the integration of emergent biomarker candidates, such as proteomic, molecular, and immunologic surrogates of vaccine-derived versus infection-derived spike, providing a baseline step towards a formal diagnostic approach of PACVS.

## Data Availability

The original contributions presented in the study are included in the article/supplementary material, further inquiries can be directed to the corresponding author.

## Footnotes

1 For the purpose of this review, we prefer the term Post-Acute COVID-19 Syndrome (PACS), given we reference the related condition, Post-Acute COVID-19 Vaccination Syndrome (PACVS). The reader may consider Long COVID (LC) and PACS to be interchangeable.
